# Tumor Cell-Specific and Lipase-Responsive Delivery of Hydrogen Sulfide for Sensitizing Chemotherapy of Pancreatic Cancer

**DOI:** 10.3389/fbioe.2022.934151

**Published:** 2022-07-11

**Authors:** Libing Tian, Rui Pei, Xiaojun Zhang, Kun Li, Yuting Zhong, Yougen Luo, Shu-Feng Zhou, Lichan Chen

**Affiliations:** ^1^ College of Chemical Engineering, Huaqiao University, Xiamen, China; ^2^ Department of Basic Medical Science, Jiangsu Vocational College of Medicine, Yancheng, China

**Keywords:** synergetic gas-chemotherapy, targeted drug delivery, pancreatic cancer, micelles, H_2_S

## Abstract

The inability of small molecule drugs to diffuse into tumor interstitium is responsible for the relatively low effectiveness of chemotherapy. Herein, a hydrogen sulfide (H_2_S) gas–involved chemosensitization strategy is proposed for pancreatic cancer treatment by developing a tumor-specific lipase-responsive nanomedicine based on aptamer-conjugated DATS/Dox co-loaded PCL-*b*-PEO micelle (DA/D@Ms-A). After receptor-mediated endocytosis and subsequent digestion of PCL blocks by intracellular lipase, the nanomedicine releases Dox and DATS, which then react with intracellular glutathione to produce H_2_S. The cytotoxicity result indicates that H_2_S can enhance Dox chemotherapy efficiency owing to the synergetic therapeutic effect of Dox and H_2_S. Moreover, the nanomedicine is featured with well tumor penetration capability benefitting from the targeting ability of aptamers and high *in vivo* biocompatibility due to the high density of PEO and biodegradable PCL. The nanomedicine capable of synergetic gas-chemotherapy holds great potential for pancreatic cancer treatment.

## Introduction

Pancreatic cancer is one of the most malignant cancers with high mortality (Sung et al., 2021) since pancreatic malignancies are difficult to diagnose in the early stage and only a few of them are resectable (Kamisawa, Wood, Itoi, & Takaori, 2016; Goess & Friess, 2018). Chemotherapy is one of the treatment choices for pancreatic cancer patients who are not surgical candidates (Kamisawa et al., 2016). Although the chemotherapeutic drugs such as gemcitabine and doxorubicin (Dox) have good anticancer activity towards pancreatic cancer, the overall chemotherapeutic outcome is sometimes not ideal due to the limitations of the small molecular drugs including short circulation half-life and poor water solubility associated nonspecific off-target effects (Lohse & Brothers, 2020). Targeted drug delivery systems provide an excellent solution to these limitations (Su, Yang, Gao, Liu, & Li, 2020; F.; Yang et al., 2011). A targeted drug delivery system usually consists of both a stimuli-responsive drug nanocarrier and a targeting moiety. The targeting moiety allows for selective accumulation of the nanocarrier at target sites, and the stimulus-response enables drug release from the nanocarrier controllable, thus achieving enhanced chemotherapeutic efficacy while reducing side effects. Many efforts have been made to develop various targeted drug delivery systems, but the chemotherapeutic efficacy is still relatively low due to the inability of small molecule drugs to diffuse into tumor interstitium.

Gas-involved cancer therapy has attracted intense attention in recent years due to its biosafety and capability to sensitize current anticancer modalities such as chemotherapy, radiotherapy, immunotherapy, photodynamic therapy, photothermal therapy, ultrasound therapy, and sonodynamic therapy (L. C. Chen, Zhou, Su, & Song, 2019; Yu, Hu, & Chen, 2018). The sensitization is usually achieved *via* the following mechanisms: 1) the capability of gas molecules to diffuse freely through the cell membrane and into the tumor depth region (Mathai et al., 2009; Riahi & Rowley, 2014), 2) inhibiting the cancer cells survival with appropriate gas concentration by reversing the Warburg effect (Szabo, 2016). Hydrogen sulfide (H_2_S), an important gasotransmitter in human health and disease, has been found to exert dose-dependent functions in cancer biology ranging from cytoprotective to cytotoxic effects (Szabó, 2007). The millimolar concentration of H_2_S promotes cancer cell apoptosis by inhibition of mitochondrial activity, activation of the endoplasmic reticulum stress response, and elevation of intracellular reactive oxygen species concentration (Szabó, 2007).

**FIGURE 1:**
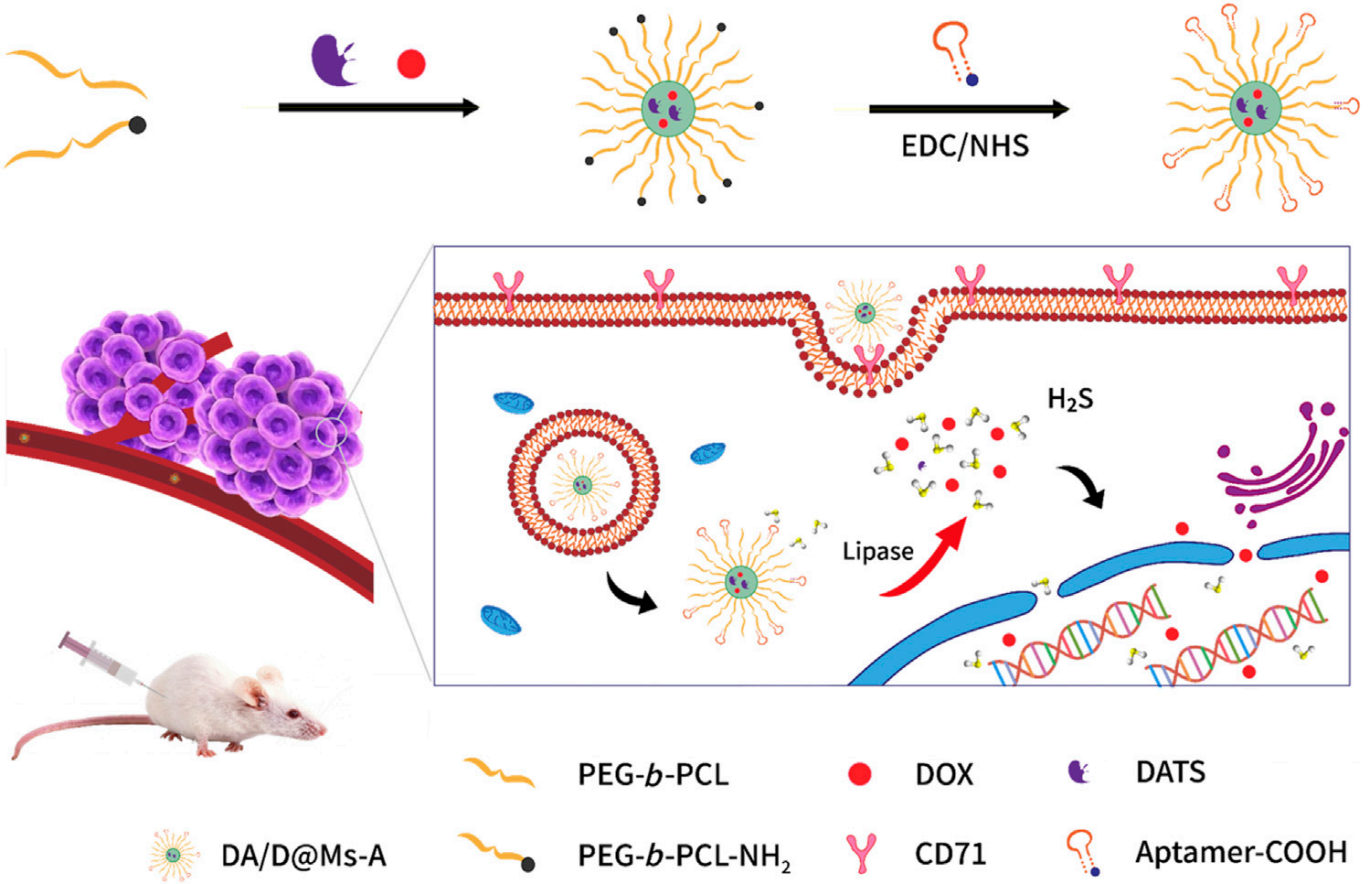
Schematic illustration of the working mechanism of aptamer targeting micelles for synergetic chemo-H_2_S gas therapy.

In this work, we hypothesize that synergetic H_2_S-chemotherapy can promote chemotherapeutic efficacy toward pancreatic cancer. To test the hypothesis, a pancreatic tumor-specific lipase-responsive nanomedicine is firstly prepared based on aptamer-conjugated small molecule chemotherapeutic drug and H_2_S prodrug coloaded micelles (Figure 1A). The micelles are constructed by self-assembly of amphiphilic diblock copolymers poly(ε-caprolactone)-block-poly(ethylene oxide) (PCL-*b*-PEO) and amino terminal PCL-*b*-PEO (PCL-*b*-PEO-NH_2_) for their FDA approval, biocompatibility and biodegradability by endogenous lipase that is highly expressed in pancreatic cancer. The co-loading of drugs for synergetic H_2_S-chemotherapy is achieved by simultaneous encapsulation of chemotherapeutic drug Dox and H_2_S prodrug diallyl trisulfide (DATS) in micelles during self-assembly of the copolymers *via* hydrophobic interaction between PCL blocks and the drugs. The surface functionalization of the micelles is realized by conjugating the carboxyl terminal XQ-2d ssDNA aptamer against CD71 (Wu et al., 2019), a transferrin receptor highly expressed on the surface of pancreatic cancer cells, onto the amino terminal of PEO blocks *via* amidation reaction. The morphology and drug-loading efficiency of the micelles are investigated followed by their targeting and penetration capability towards pancreatic cancer cells and multicellular spheroids. By treating pancreatic cancer cells with the micelles, the micelles enter into the cells by CD71 receptor-mediated endocytosis, and the PCL blocks in the micelles are digested by intracellular lipase to release Dox and DATS (Li et al., 2002), which then reacts with intracellular glutathione to produce H_2_S (Liang, Wu, Wong, & Huang, 2015) (Figure 1B). The therapeutic efficacy of the micelles evaluated by the cell counting kit-8 (CCK-8) assay demonstrates that H_2_S indeed enhances the chemotherapeutic efficacy of Dox in pancreatic cancer cells. The biocompatibility of the micelles is further analyzed in healthy mice. The nanomedicine capable of synergetic gas-chemotherapy holds a great promising prospect for improving tumor therapeutic efficacy.

## Materials and Methods

### Materials

Dimethyl sulfoxide (DMSO), triethylamine (TEA), N-hydroxy-succinimide (NHS), N-ethyl-N′-(3-dimethyl-aminopropyl) carbodiimide (EDC), and diallyl trisulfide (DATS) were purchased from Alfa Aesar. Lipase from *Pseudomonas cepacia* (38.6 U/mg) was bought from Sigma. The amphiphilic diblock copolymers poly(ε-caprolactone)-block-poly(ethylene glycol) without and with a functional amino group, PCL_5k_-*b*-PEO_5k,_ and PCL_5k_-*b*-PEO_5k_-NH_2_, were obtained from Ruixi Bio-Tech Co., Ltd. (Xi’an, China). Dialysis bags with various molecular weights cut off were from Viskase. Human pancreatic cancer cells (Panc-1) and human liver cancer cells (HepG2) were ordered from Shanghai Institute of Biological Sciences, Chinese Academy of Sciences. Phosphate buffer solution (PBS), cell culture medium (dulbecco’s modified eagle medium, DMEM), x100 penicillin-streptomycin solution, Trypsin/EDTA solution, and hematoxylineosin (HE) were purchased from Shanghai Peiyuan Bio-Tech Co., Ltd. Fetal calf serum (FBS) was received from Gibco Company (United States). Doxorubicin (DOX), cell counting kit-8, diaminophenylindole (DAPI), Hoechst 33258, propidium iodide (PI), and annexin V-FITC were procured from Solarbio (Beijing, China). Washington State Probe-1 (WSP-1) for fluorescence imaging of H_2_S release in living cells was from Maokang Bio-Tech Co. Ltd. (Shanghai, China). KM mice (SPF grade, 25 g, half male and half female) were ordered from the Fuzhou Wushi laboratory animal center (Fujian, China). All chemicals were used as received. Ultrapure water with a resistivity of 18.2 MΩ (Millipore) was used throughout this work. The carboxyl modified XD-2d aptamer against CD71 was obtained from Sangon Biotech (Shanghai, China), and its base sequence was shown as follows: 5′-COOH-ACT CAT AGG GTT AGG GGC TGC TGG CCA GAT ACT CAG ATG GTA GGG TTA CTA TGA GC-3′.

### Characterizations

Transmission electron microscopy (TEM) images of micelles were acquired on a Japan Hitachi HT7700 electronic microscope by negatively staining the micelles with 2.0% phosphotungstic acid. The hydrodynamic diameter and zeta potential of micelles were determined using a dynamical light scattering (DLS) system (NanoBook Omni Instrument, United States). UV-vis spectra were recorded with a UV-2450 UV-vis spectrometer (Shimadzu, Japan). Fluorescent images were captured on a Leica TCS SP8 confocal laser scanning microscopy (CLSM) (Leica, Germany). Cell apoptosis was measured by a BD FACS Melody flow cytometer.

### Preparation of Aptamer-Conjugated Drug-Loaded Micelles

1 ml DMSO was subsequently added with 8 mg PCL-*b*-PEO, 2 mg PCL-*b*-PEO-NH_2_, 5 mg DOX and/or 5 mg DATS, and 10 μl TEA for neutralization. The solution was stirred at room temperature for 4 h. After complete dissolution, the solution was dropwise added into 10 ml water with gentle stirring for 8 h to allow the formation of drug-loaded micelles. The solution was then dialyzed using cellulose dialysis membranes (MW cut off ∼14 kDa) against water for 3 days to remove the residual organic solvent and free doxorubicin and/or DATS. The as-obtained micelles were filtrated against a 0.22 μm filter membrane and stored in a 4°C refrigerator for later use. The blank micelles, DOX-loaded micelles, DATS-loaded micelles, and DOX and DATS co-loaded micelles were designated as BMs, D@Ms, DA@Ms, and DA/D@Ms, respectively.

The micelles were further conjugated with the aptamer *via* an amidation reaction (Tian, Pei, Zhong, Ji, & Zhou, 2020). Briefly, 1 mg EDC, 1 mg NHS, and 10 OD carboxyl modified aptamer were dissolved in 5 ml water and reacted for 2 h to activate the carboxyl group of the aptamer, then added with the micelles above and reacted for 72 h. The aptamer-conjugated micelles were obtained after dialysis using cellulose dialysis membranes (MW cut off ∼20 kDa) against water for 3 days. Aptamer-conjugated DOX and DATS co-loaded micelles were designated as DA/D@Ms-A. The stability of the micelles was investigated by measurement of their zeta potentials after suspending in PBS and full medium (DMEM supplemented with 10% FBS and 1% penicillin-streptomycin) for 48 h.

The drug loading efficiency was defined as the weight percentage of the drug in the micelles and quantified by measuring the absorbance of DOX at 480 nm and DATS at 354 nm using a UV-vis spectrophotometer. Typically, the freeze-dried drug-loaded micelles were dissolved in DMSO for UV-vis measurement. The drug content was determined by the calibration curve, which was obtained with various concentrations of DOX or DATS in the DMSO solution.

### *In Vitro* Lipase Responsive Drug Release From the Micelles

The stimuli-responsive release profiles of drugs from the micelles were studied at 37°C in PBS with different concentrations of lipase. Briefly, 2 ml 1 mg/ml D@Ms in 10 mM pH 7.4 PBS with 0.2 mg/ml and 1 mg/ml lipase was transferred into two dialysis bags (MW cut off ∼10 kDa), which were then immersed into 4 ml of the release media (10 mM pH 7.4 PBS), respectively. At selected time intervals, the release media was removed for UV-vis analysis and replaced with fresh release media. Dox concentration was calculated based on the absorbance intensity at 480 nm. In the release profiles describing drug-release behavior, the cumulative release percentages of drugs from micelles were plotted against time. The percentage of cumulative release was calculated according to the formula: cumulative release percentage (%) $=2\times \frac{\Sigma_{C_{n}}}{W_{0}}\times 100\%$ , where *C*_n_ and W_0_ refer to the Dox concentration of the n time collected release media and the total Dox amount in micelles. The release experiments were conducted in triplicate.

### Fluorescence Imaging of H_2_S Release From the Micelles in Living Cells

Panc-1 cells were seeded in a 35 mm confocal dish at 1.0×10^5^ cells per well, and cultured with a full medium in a humidified atmosphere of 5% CO_2_ at 37°C. When the cells grew to about 70%, the medium was removed, and a 1.0 ml fresh serum-free medium containing 0.01 mg/ml of DA@Ms and DA@Ms-A were then added and incubated for 2 and 4 h, respectively. After that, the cells were washed with PBS, incubated with a 15 μM WSP-1 probe in PBS for 40 min, then washed with PBS three times and added with 1 ml PBS. The generation of H_2_S was observed immediately by CLSM with an excitation wavelength fixed at 488 nm.

### Selective Cellular Uptake and Penetration Capability of the Micelles

Panc-1 cells and HepG2 cells were seeded in a 35 mm confocal dish at 1.0×10^5^ cells per well, and cultured with a full medium at a humidified atmosphere of 5% CO_2_ at 37°C. When the cells grew to about 70%, the medium was removed, and a 1.0 ml fresh serum-free medium containing 0.01 mg/ml of DA@Ms and DA@Ms-A were then added and incubated for 0.5, 1, and 2 h, respectively. After that, the cells were stained with DAPI according to the instructions provided by the manufacturer, washed with PBS three times, and added with 1 ml PBS. The targeting efficiency of the micelles was observed immediately by CLSM with excitation wavelength fixed at 488 nm for the Dox channel and 405 nm for the DAPI channel.

3D cell spheroids were cultured to investigate the penetration capability of the micelles. In brief, 10 ml of 1.5 w/v% hot agarose solution was added to a 25 ml dish. After cooling down, the semisolid agarose coating provided a non-adherent surface preventing cellular adhesion. 1.0×10^6^ Panc-1 cells in 15 ml full medium was transferred to the dish, and cultured for 4 days to generate spheroids. The spheroids were collected by centrifugation, washed with PBS, and resuspended in a 1 ml serum-free medium containing 0.01 mg/ml DA/A@Ms and DA/D@Ms-A, respectively. After incubation for 4, 12, and 24 h, the spheroids were washed with PBS three times and then observed by CLSM Z-stack scanning with excitation wavelength fixed at 488 nm.

### Cytotoxicity Evaluation

Panc-1 cells were seeded in 96-well plates with 1×10^4^ cells per well, and cultured with a full medium for 24 h. Then the cells were treated with DMEM medium containing 0.1 mg/ml BMs, D@Ms, DA@Ms, DA/D@Ms, DA/D@Ms-A and Dox for 72 h, 0.05 mg/ml Dox for 36 h then 0.05 mg/ml DATS for 36 h, and 0.05 mg/ml DATS for 36 h then 0.05 mg/ml Dox for 36 h, respectively. Cell viability was determined with CCK-8 assay according to the instructions provided by the manufacturer. The cell viability was calculated according to the formula: cell viability $=\frac{OD_{450}^{Treat\;group}-OD_{450}^{Blank\;group}}{OD_{450}^{Control\;group}-OD_{450}^{Blank\;group}}$ ×100%.

To test apoptosis by Hoechst 33258, Panc-1 cells were seeded in a 35 mm confocal dish at 1.0×10^6^ cells per well, cultured with a full medium for 24 h, and then cultured with serum-free medium containing 0.1 mg/ml micelles for 24 h. The cells were stained by Hoechst 33258 for 30 min, washed three times with PBS, added with 1 ml PBS, and then observed by CLSM with excitation wavelength fixed at 405 nm.

To quantitate apoptosis, Panc-1 cells were seeded in 12-well plates with 1.0×10^6^ cells per well, cultured with a full medium for 24 h, and then treated with serum-free medium containing 0.1 mg/ml micelles for 24 h. The collected cells were washed twice with cold PBS and then resuspended in 0.5 ml PBS. The cells were then co-stained by PI and annexin V-FITC and analyzed by flow cytometry.

### Biosafety Evaluation of the Micelles

Forty-eight mice were divided into six groups, including a control, BMs, DA@Ms, D@Ms, DA/D@Ms, and DA/D@Ms-A group. The samples (0.1 ml of 1 mg/ml micelle suspension or sterile PBS as control) were injected into the abdominal cavity of each mouse every other day. The body weight of mice was recorded every other day, and the clinical manifestations and fatalities were observed daily. After 2 weeks, the heart, liver, spleen, lung, and kidney were dissected, rinsed with PBS, fixed in 4% paraformaldehyde, then subjected to paraffin section, HE staining, and histopathological examination.

## Results and Discussion

### Preparation and Characterization of Aptamer-Conjugated Drug-Loaded Micelles

**FIGURE 2:**
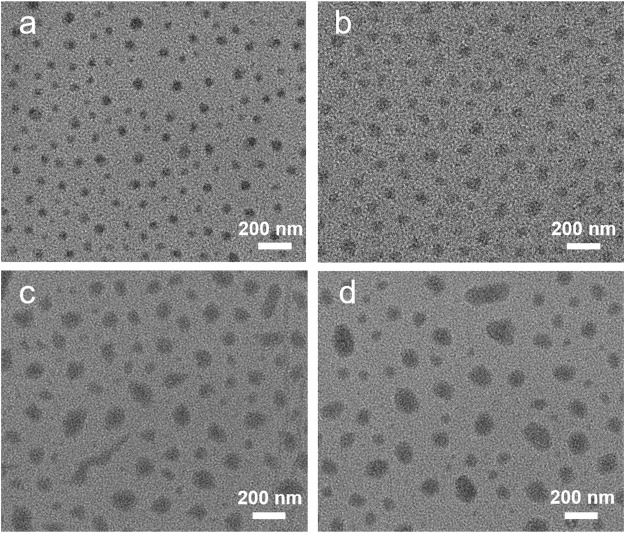
TEM images of **(A)** D@Ms, **(B)** DA@Ms, **(C)** DA/D@Ms, and **(D)** DA/D@Ms-A.

The drug-loaded micelles were prepared by simultaneous encapsulation of Dox and DATS in micelles during self-assembly of the diblock copolymers PCL-*b*-PEO and PCL-*b*-PEO-NH_2_. The aptamer functionalization of the drug-loaded micelles was realized by the covalent binding of the carboxyl terminal XD-2d aptamer to the amino terminal micelles through an amidation reaction. The hydrodynamic size of the micelles was characterized by DLS measurement. As shown in Table 1, the hydrodynamic size of blank micelles (BMs) is ca. 87.68 nm; after drug loading, the sizes of the micelles are increased to 178.83 nm for Dox loaded micelles (D@Ms), 176.68 nm for DATS loaded micelles (DA@Ms), and 195.95 nm for DATS and Dox coloaded micelles (DA/D@Ms). The micelles size increment after drug loading is because the hydrophobic DOX and DATS change the hydrophobic bonds in the micellar core and thus increase the packing density and the size of the hydrophobic micellar core. Notably, after aptamer conjugation to the surface of DA/D@Ms through amidation reaction, the size of the micelles is further increased to 209.80 nm, indicating the successful formation of aptamer modified DA/D@Ms (DA/D@Ms-A). The size distribution and morphology of the micelles were further investigated by TEM. As shown in Figure 2, the micelles with and without drug loading are near-spherical in shape and are monodisperse particles with high uniformity; and the particle sizes are gradually increased after drug loading and aptamer conjugation, that is, 76, 75, 146, and 163 nm for D@Ms, DA@Ms, DA/D@Ms, and DA/D@Ms-A, respectively, agreeing well with the results obtained from DLS measurement. The sizes of the micelles obtained from TEM are smaller than those obtained from DLS measurement. This is because the collapse in the hydrophilic layer of the micelles during TEM sample preparation leads to reduced particle sizes of the micelles.

**TABLE 1:** Characterization of the micelles.

Micelles	DLS (nm)	Zeta potential (mV)			Drug-loading efficiency (%)
		H_2_O^a^	PBS^a^	Full medium^a^ ^,^ ^b^	
BMs	87.68 ± 3.52	−18.74	−12.90	−13.35	-
D@Ms	178.83 ± 4.07	−20.74	−14.40	−15.91	10.34
DA@Ms	176.68 ± 5.48	−19.19	−14.99	−14.28	9.06
DA/D@Ms	195.95 ± 5.89	−21.88	−15.75	−16.58	16.32
DA/D@Ms-A	209.80 ± 6.07	−23.73	−16.80	−30.48	17.83

a Zeta potential was measured after suspending the micelles in the solvents for 48 h. All data presented were the average of three parallel assays.

b Full medium: DMEM supplemented with 10% FBS and 1% penicillin-streptomycin.

The change in the zeta potential of the micelles was also recorded to monitor the surface decoration. As shown in Table 1, the zeta potential of the micelles in ultrapure water shows a slight change after drug loading but significantly increases after aptamer conjugation, indicating that aptamer modification could increase the stability of the micelles. To examine their stability in different media, the micelles were kept in PBS and full medium for 48 h followed by zeta potential measurement. The slight change indicates good stability of the micelles in PBS and full medium. Furthermore, it can be seen from Table 1 that simultaneous encapsulation of DATS and Dox in micelles can greatly increase the drug loading efficiency of the micelles, providing the possibility of higher therapeutic efficiency.

### Selective Cellular Uptake and the Penetration Capability of the Micelles

**FIGURE 3:**
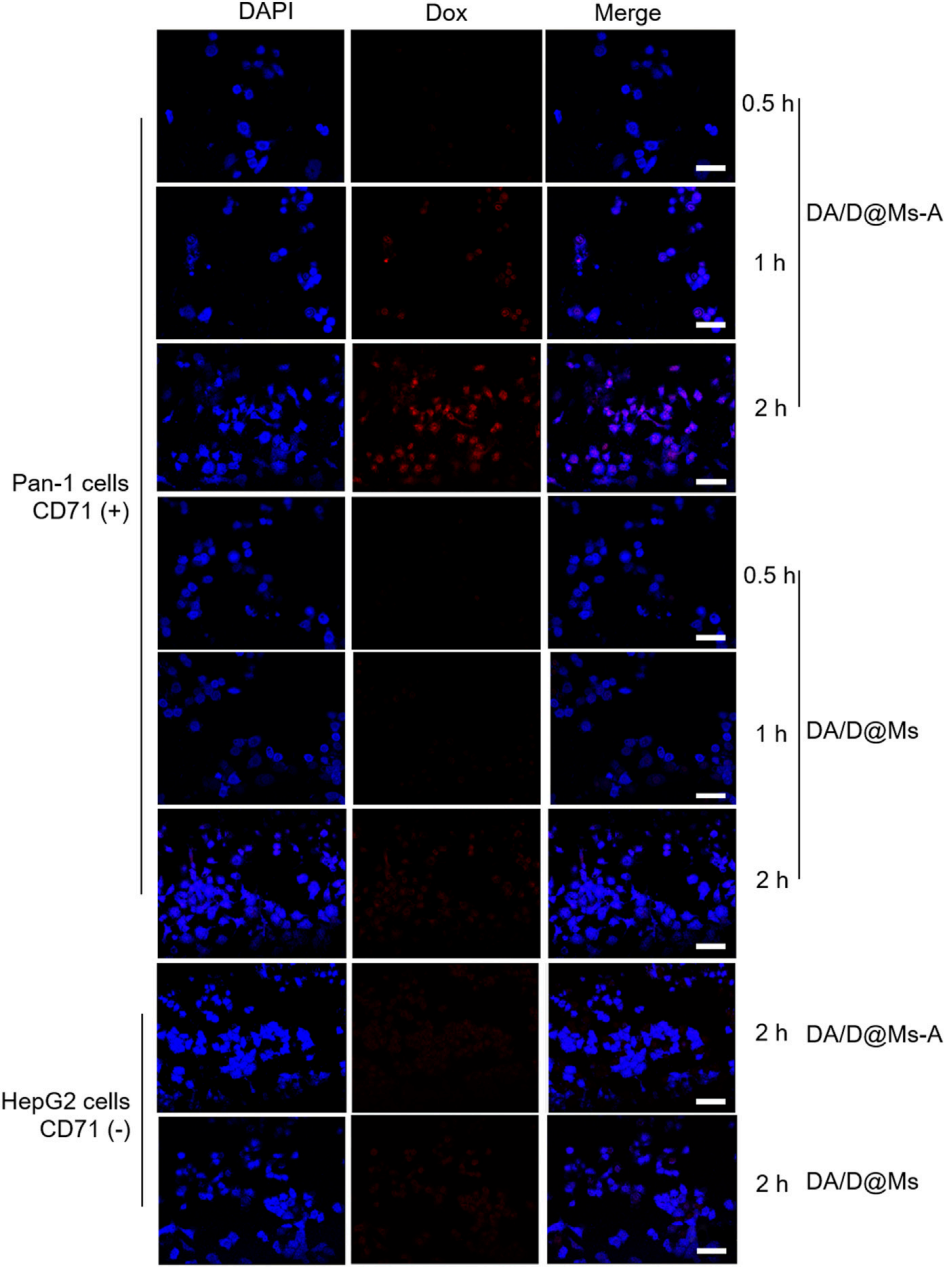
CLSM images of Panc-1 cells and HepG2 cells incubated with 0.01 mg/ml DA/D@Ms-A and DA/D@Ms for 0.5, 1, and 2 h, respectively. DAPI channel with excitation wavelength at 405 nm exhibits blue fluorescence from DAPI stained cell nuclei. Dox channel with excitation wavelength at 488 nm shows red fluorescence from Dox. Scale bar = 50 μm.

Selective cellular uptake of therapeutic drugs is important for cancer therapy to reduce side effects. Specific ligand-mediated targeting is a feasible strategy to realize selective cellular uptake. To examine the selectivity, we compared the internalization of DA/D@Ms-A and DA/D@Ms in Panc-1 cells overexpressing CD71 and HepG2 cells that hardly express CD71. The cells seeded in confocal dishes were incubated with 0.01 mg/ml DA/D@Ms-A and DA/D@Ms for different time, that is, 0.5, 1, and 2 h, and the cellular uptake was monitored by checking the fluorescence intensity of Dox using CLSM. As shown in Figure 3, Panc-1 cells incubated with DA/D@Ms-A exhibit bright red fluorescence in the Dox channel, and the fluorescence intensity increases with time, indicating the effective accumulation of the micelles within the cells by aptamer-receptor mediated endocytosis; while Panc-1 cells incubated with DA/D@Ms and HepG2 incubated with both DA/D@Ms-A and DA/D@Ms display weak red fluorescence in Dox channel, suggesting that only a few micelles enter into the cells by pinocytosis. All the results indicate that aptamer modification endows the micelles with high targeting efficiency and good selectivity towards pancreatic cancer cells with CD71 overexpression.

**FIGURE 4:**
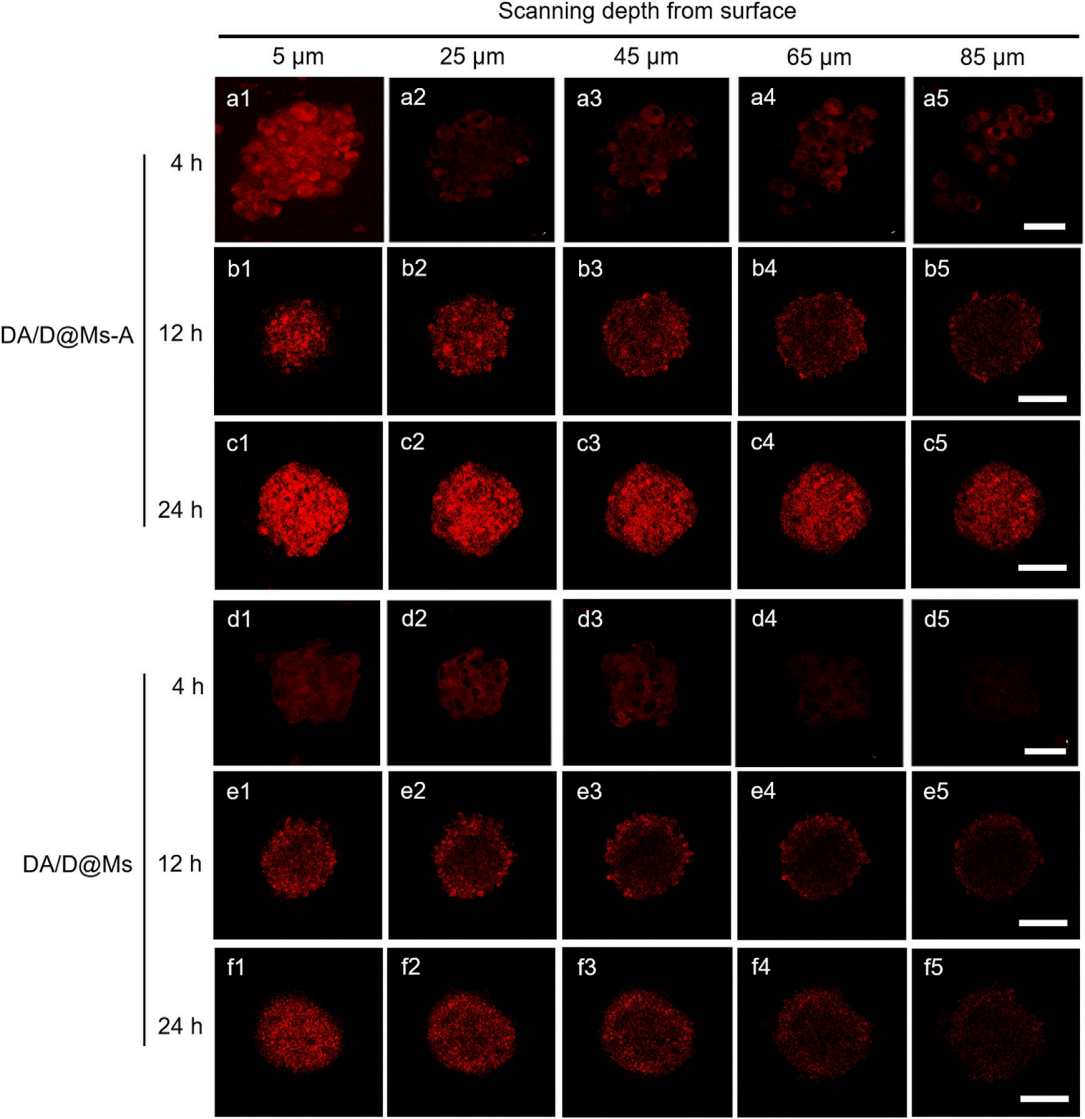
CLSM images showing *in vitro* penetration of DA/D@Ms-A and DA/D@Ms in Panc-1 multicellular spheroids. The spheroids were incubated with 0.01 mg/ml DA/D@Ms-A or DA/D@Ms for 4, 12, and 24 h, respectively, and measured by CLSM Z-stack scanning. The surface of the spheroids was defined as 0 μm. Excitation wavelength = 488 nm, scale bar = 50 μm.

In addition to targeting efficiency, the penetration capability of the micelles was also investigated by CLSM tomography using Panc-1 cell-derived multicellular spheroids (MCSs) as an *in vitro* model. MCSs are versatile three-dimensional models for cancer theranostics benefiting from their similarity in morphology and biological microenvironment to solid tumors (Abbott, 2003). The penetration activity of DA/D@Ms-A and DA/D@Ms was monitored by CLSM Z-stack scanning after incubation of MCSs with 0.01 mg/ml DA/D@Ms-A and DA/D@Ms for different time, that is, 4, 12, and 24 h. As shown in Figure 4, strong red fluorescence from Dox can be observed on the periphery of the MCSs incubated with DA/D@Ms-A for 4 h due to the targeting capability of DA/D@Ms-A towards the Panc-1 MSCs. The red fluorescence in the interior area of the MSCs can clearly be observed even at the scanning depth of 85 μm. In the case of DA/D@Ms for 4 h, red fluorescence can be seen on the periphery of the MCSs due to the enhanced permeation and retention effect mediated passive targeting of DA/D@Ms, however, the red fluorescence in the interior area of the MSCs rapidly attenuates and becomes faint at the scanning depth of 65 μm. The results indicate that the aptamer modification endows DA/D@Ms-A with great tumor penetration capability. It is worth noting that with the increase in incubation time, the red fluorescence brightness increases in both the cells incubated with DA/D@Ms-A and DA/D@Ms, however, the brightness of the red fluorescence enhances greatly even at the scanning depth of 85 μm after incubation of Panc-1 MCSs with DA/D@Ms-A for 24 h. This is probably because that cell apoptosis induced by DA/D@Ms-A causes the enhancement in permeability of cell membrane and MSCs, which in turn facilitates the penetration of DA/D@Ms-A in MSCs.

### *In Vitro* Cytotoxicity of the Micelles

**FIGURE 5:**
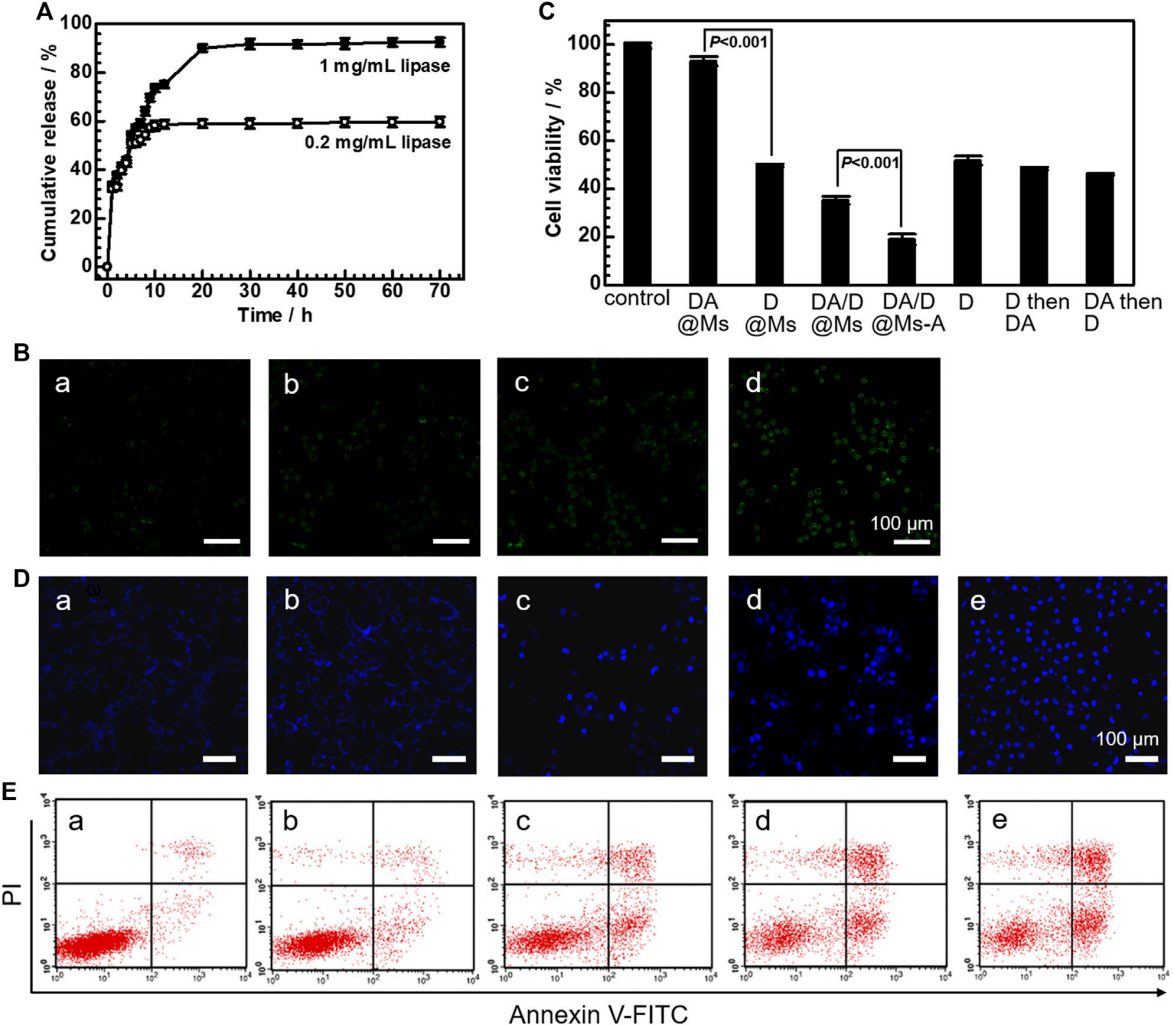
*In vitro* cytotoxicity. **(A)** The release profiles of Dox from D@Ms in the presence of 0.2 mg/ml and 1 mg/ml lipase. **(B)** Fluorescence imaging of H_2_S release in Panc-1 living cells after incubation with **(a,b)** DA@Ms and **(c,d)** DA@Ms-A for 2 h **(a,c)** and 4 h **(b,d)**, respectively. **(C)** Cell viability of Panc-1 cells treated with 0.1 mg/ml micelles and Dox for 72 h, 0.05 mg/ml Dox for 36 h then 0.05 mg/ml DATS for 36 h, and 0.05 mg/ml DATS for 36 h then 0.05 mg/ml Dox for 36 h by CCK-8 assay, respectively. **(D)** CLSM images of Panc-1 cells stained with Hoechst 33258 and **(E)** flow cytometry of Panc-1 cells stained with FTIC and PI after treatment with 0.1 mg/ml micelles for 24 h. **(a)** control, **(b)** DA@Ms, **(c)** D@Ms, **(d)** DA/D@Ms, and **(e)** DA/D@Ms-A.

The lipase-responsive drug release profile from the micelles was investigated by taking D@Ms as an example. Figure 5A shows the release profile of Dox from D@Ms in PBS containing 0.2 mg/ml and 1 mg/ml lipase at 37°C. In the presence of 0.2 mg/ml lipase, 50.68% of Dox was released in 5 h and 58.96% was released in 20 h. With a higher lipase concentration of 1 mg/ml, 54.31% of Dox was released in 5 h and 90.00% was released in 20 h. The results confirm the possibility of lipase-responsive drug release from micelles by lipase-mediated PCL blocks digestion-induced micelles disassembly.

To verify the intracellular glutathione-triggered H_2_S release, a commercially available probe (WSP-1), whose fluorescence can be selectively turned on by H_2_S (Liu et al., 2011), was employed for the detection of H_2_S in living Panc-1 cells. As shown in Figure 5B, Panc-1 cells exhibit weak fluorescence of WSP-1 after incubation with DA@Ms for 2 h, and the fluorescence becomes brighter with the increase of incubation time. Panc-1 cells display much stronger fluorescence of WSP-1 after incubation with DA@Ms-A, indicating that more H_2_S is generated in cells by taking advantage of the targeting capability of DA@Ms-A. The results suggest that after the micelles enter into cells, the digestion of PCL blocks by intracellular lipase leads to the release of the encapsulated DATS from the hydrophobic micellar core into the cytoplasm, and the DATS subsequently reacts with intracellular glutathione to generate H_2_S.

To evaluate the therapeutic efficacy of the micelles, the cell viability was monitored by CCK-8 assay after incubation of Panc-1 cells with the micelles for 72 h. As shown in Figure 5C, BMs have no obvious cytotoxic effect against Panc-1 cells, indicating the biocompatibility of the micelles assembled by diblock copolymers PCL-*b*-PEO and PCL-*b*-PEO-NH_2_. The cell viability of Panc-1 cells treated with DA@Ms, D@Ms, and DA/D@Ms is decreased to 93.11%, 49.73%, and 35.19%, respectively. This suggests that the therapeutic effect of DATS is not significant when it is used alone, but it can act as a sensitizer to enhance the therapeutic effect of Dox by combined DATS and Dox treatment. The micelles such as DA@Ms, D@Ms, and DA/D@Ms enter into cells by pinocytosis and release the encapsulated Dox and DATS, which then reacts with intracellular glutathione to generate H_2_S. H_2_S with a concentration above a certain value has been reported to exhibit a cytotoxic effect by inducing intense intracellular acidification *via* overdriving cancer glycolysis (Cao et al., 2019), causing apoptosis *via* downregulation of the downstream anti-apoptotic proteins (Kashfi, 2014; Yao, Yang, Xu, He, & Yang, 2022), inducing mitochondrial dysfunction *via* suppression of the mitochondrial cytochrome c oxidase activity (Hill et al., 1984; Szabó, 2007; Szabo et al., 2014; Z. Yang et al., 2021), and aggravating hypoxia and oxidative stress *via* suppression of catalase expression (Xie et al., 2020). H_2_S has also been found to be able to activate hypoxia-responsive prodrugs (Fang et al., 2020), and to upregulate the level of intracellular Dox by decreasing P-gp mediated efflux (Chegaev et al., 2016), thus promoting the chemotherapeutic efficacy. Therefore, it can be deduced that the achieved H_2_S-enhanced chemotherapy in this work has resulted from the intrinsic cytotoxicity of H_2_S, the facilitation of Dox release from the hydrophobic micellar core into the cytoplasm due to the intense intracellular acidification caused by H_2_S and the pH-related release property of Dox, and the accumulation of Dox inside cells owing to the less efflux. The chemosensitization of H_2_S is furthered improved by the comparable therapeutic efficacy of Panc-1 cells treated with 0.1 mg/ml Dox only, 0.05 mg/ml Dox then 0.05 mg/ml DATS, and 0.05 mg/ml DATS then 0.05 mg/ml Dox, namely 51.70%, 48.45% and 46.00%, respectively. The cell viability is further down to 18.41% by incubation of Panc-1 cells with DA/D@Ms-A. This suggests that the aptamer-CD71 receptor interaction mediated endocytosis promotes the cancer cell-killing efficacy by increasing the intracellular drug concentration. The higher cytotoxicity reached by the lower drug concentration as compared to the free Dox provides DA/D@Ms-A a promising prospect in raising therapeutic effects and reducing side effects in clinical application.

Apoptosis is the main mechanism accounting for the anticancer action of Dox and H_2_S. Apoptotic cell death was confirmed by nucleus chromatin condensation by Hoechst 33258 staining. As shown in Figure 5D, the nuclei of Panc-1 cells treated with BMs can only stain an ultraweak and homogeneous blue color since Hoechst 33258 is unable to infiltrate into the living cells, while the nuclei of Panc-1 cells treated with DA@Ms or D@Ms stains bright blue due to the synergetic effect of apoptosis associated cell membrane permeability enhancement and apoptosis induced chromatin condensation. The blue emission light in Panc-1 cells treated with DA/D@Ms is much bright than those treated with DA@Ms and D@Ms only, suggesting that more cells undergo apoptosis by the strong synergetic effects between Dox and H_2_S. The brightness of the blue emission light in Panc-1 cells treated with DA/D@Ms-A is further enhanced than those treated with DA/D@Ms, indicating that the targeting strategy promotes the therapeutic efficacy of synergetic gas and chemotherapy. To further examine the apoptotic characteristics in the micelles treated Panc-1 cells, flow cytometry was used to investigate the treated cells co-stained with Annexin V-FTIC and PI. Annexin V-FITC is a phospholipid-binding protein with a high affinity for phosphatidylserine, which can be used as a sensitive probe for phosphatidylserine exposure to the cell membrane (S. Chen, Cheng, Wang, & Peng, 2008). During the early apoptosis, the cells become reactive with annexin V-FITC after the onset of chromatin condensation, but prior to the loss of the plasma membrane's ability to exclude PI. On the basis, non-apoptotic cells, early apoptotic cells, late apoptotic/necrotic cells, and dead cells can be discriminated by co-staining the cells with annexin V-FITC and PI. As shown in Figure 5E, after incubation with BMs, DA@Ms, and D@Ms for 24 h, the percentage of apoptotic cells was 3.53%, 9.73%, and 30.35%, respectively. The percentage of apoptotic cells increases to 40.97% by treatment with DA/D@Ms, and further increases to 50.18% by treatment with DA/D@Ms-A, verifying the effectiveness of targeted synergetic gas-chemotherapy.

### *In Vivo* Biocompatibility of the Micelles

**FIGURE 6:**
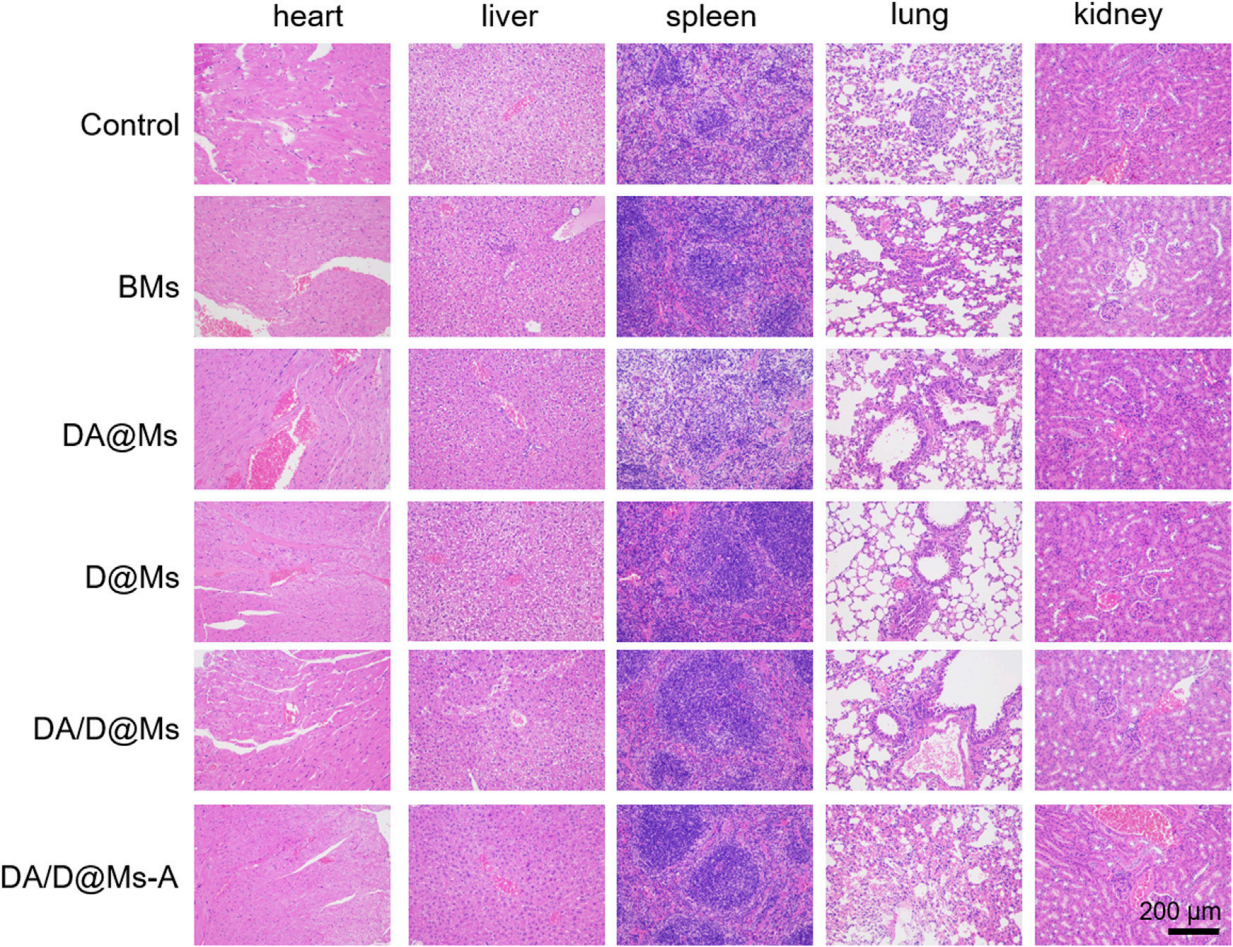
HE stained major organs collected from mice post intraperitoneal administration of micelles every other day for 2 weeks. Scale bar = 200 μm.

The *in vitro* toxicity of the micelles was evaluated by intraperitoneal administration of 0.1 ml 1 mg/ml micelles to healthy mice every other day for 2 weeks. HE staining was used to investigate morphological changes in the major organs such as the heart, liver, spleen, lung, and kidney in mice. The results are shown in Figure 6. All the tissue slices display normal cell morphology, well-organized tissue structures, and no significant lesions, indicating that the micelles are biocompatible, and safe for clinical applications.

## Conclusion

In summary, we have proposed an H_2_S gas-involved chemosensitization strategy for improving pancreatic cancer chemotherapeutic efficacy. The nanomedicine for synergetic gas-chemotherapy has been constructed by encapsulation of both Dox and DATS in aptamer conjugated PCL-*b*-PEO micelles. The DA/D@Ms-A nanomedicine with the size of *ca.* 200 nm has shown well colloidal stability, competitive drug loading efficiency, effective targeting efficiency, and well penetration capability towards CD71 overexpressed multicellular spheroids, good *in vitro* and *in vivo* biocompatibility, and endogenous lipase and glutathione responsiveness. The DA/D@Ms-A nanomedicine with a concentration of 0.1 mg/ml was able to kill *ca.* 82% of pancreatic cancer cells after 72 h incubation, and the therapeutic efficiency of synergetic gas-chemotherapy was *ca.* 15% higher than that of individual chemotherapy. The gas-involved chemosensitization strategy and the as-developed targeted stimuli-responsive nanomedicine hold great promise for the improvement of pancreatic cancer treatment.

## Data Availability

The original contributions presented in the study are included in the article/supplementary material; further inquiries can be directed to the corresponding authors.
